# Genetic mapping and validation of QTL for whitefly resistance in cassava (*Manihot esculenta* Crantz)

**DOI:** 10.1007/s00122-025-04949-1

**Published:** 2025-06-24

**Authors:** Adriana Bohorquez-Chaux, Luis Augusto Becerra Lopez-Lavalle, Vianey Barrera-Enriquez, María Isabel Gómez-Jiménez, Camilo E. Sanchez-Sarria, Luis Fernando Delgado, Xiaofei Zhang, Winnie Gimode

**Affiliations:** 1 Alliance of Bioversity International and the International Center for Tropical Agriculture (CIAT), Km 17, Recta Cali-Palmira, A. A. 6713 Cali, Colombia; 2 International Center for Agricultural Research in the Dry Areas (ICARDA), BP 6202, Rabat, Morocco; 3 European Bioinformatics Institute (EMBL-EBI), Welcome Genome Campus, Hinxton, Cambridge, CB10 1SD UK; 4 University of California Davis, Davis, CA 95616 USA

## Abstract

**Key message:**

QTL associated with whitefly resistance were identified in a cassava F_2_ population and KASP markers applicable in selection for the trait were validated.

**Abstract:**

Whitefly species pose a major threat to cassava production in tropical regions causing direct plant damage and transmitting viruses that lead to devastating cassava diseases. *Aleurotrachelus socialis* whitefly is one of the pests that affect cassava in South America. Developing resistant cassava varieties is the most sustainable control strategy for managing whiteflies. This study aimed to map the quantitative trait loci (QTL) associated with resistance to *A. socialis* and develop molecular markers to facilitate marker-assisted selection. An F_2_ cassava population (*N* = 183) was generated by selfing a highly resistant F_1_ derived from a cross between ECU72 (resistant) and COL2246 (susceptible) landraces. Phenotyping was performed using an efficient glasshouse screening method and high throughput image analysis of infested leaves (Nymphstar). We identified QTL on chromosomes 1, 2, 5, 6, 8, 9, and 14, with a stable and highly significant QTL on chromosome 8 (*MeF2WFly8.1*), explaining 35.44% of the phenotypic variation. To enable efficient selection, high-throughput KASP markers were developed and validated across diverse genetic backgrounds. Three SNPs displayed the highest association with whitefly resistance, with Chr08_6483145 as the most effective marker for selection in diverse backgrounds. These markers are provided for improving the efficiency of whitefly resistance breeding in the global cassava community.

**Supplementary Information:**

The online version contains supplementary material available at 10.1007/s00122-025-04949-1.

## Introduction

Whiteflies are a major threat to cassava (*Manihot esculenta* Crantz) production, not only causing direct plant damage but also transmitting viruses responsible for cassava mosaic disease (CMD) and cassava brown streak disease (CBSD) ​(Colvin et al. 2004; Maruthi et al. 2005)​. While these pests pose a significant challenge in many cassava growing regions, they are particularly devastating in sub-Saharan Africa, where frequent super-abundant whitefly outbreaks exacerbate the spread ​(Otim-Nape et al. 2001; Alicai et al. 2007​)​. In addition to significant yield losses caused by the spread of cassava diseases, whiteflies, especially in the Neotropics, contribute to direct damage by feeding on the leaf phloem, leading to symptoms such as chlorosis and premature leaf drop ​(Bellotti and Arias 2001; Bellotti et al. 2012)​. They also excrete sticky and sugary substances that coat the foliage and serve as a substrate for sooty mold ​(Bellotti and Arias 2001; Nelson 2008)​, resulting in a reduction in photosynthesis and subsequently in root yield, if the infestation is prolonged.

The most damaging whitefly species in Africa is *Bemisia tabaci,* which includes more than 40 morphologically indistinguishable putative whitefly species distributed worldwide (​de Moya et al. 2019; Mugerwa et al. 2021)​. The damage caused by the *B. tabaci* species continues to increase, with the areas affected with CBSD also rapidly expanding ​(MacFadyen et al. 2018)​. In the Americas, eleven whitefly species have been reported affecting cassava ​(Bellotti et al. 1999), with *Aleurotrachelus socialis* Bondar reported to cause significant yield losses of up to 79% ​(Vargas & Bellotti, 1981; Farias 1994; Bellotti et al. 1999) primarily through direct plant damage. Although the *B. tabaci* complex is well known for transmitting many plant viruses, *A. socialis* has not generally been considered a major virus vector. However, past research indicated *A. socialis* could transmit cassava virus X (Angel et al. 1987, 1989). Furthermore, it is hypothesized that *A. socialis* may act as an aerial vector for cassava frog skin disease (CFSD) in South America, recently established to be caused by torradoviruses (Jimenez et al. 2024), but its involvement in CFSD transmission is currently under investigation.

The control of whitefly populations in the fields relies largely on integrated pest management (IPM) measures, including cultural practices and chemical controls ​(Bellotti and Arias 2001; Carabalí et al. 2010)​. The best strategy, which is a cheaper and more environmentally sustainable option for management, would be to utilize cassava varieties with host resistance against the pest. Screening of the germplasm collection at the International Center for ​​Tropical Agriculture (CIAT) has identified donor lines with whitefly resistance ​(Bellotti et al. 1987; Parsa et al. 2015; Bohorquez-Chaux et al. 2023; Atim et al. 2024), with some sources including ECU72 exhibiting broad resistance not only against *A. socialis,* but also against various species of *Bemisia tabaci* (Omongo et al. 2012; Atim et al. 2024)*.*

Quantitative trait loci (QTL) associated with whitefly resistance have been described in other crops including tomato, soybean, melon, cotton and cabbage ​(Nombela and Muñiz 2010; Boissot et al. 2010; Xu et al. 2011; Broekgaarden et al. 2018; Aslam et al. 2023​)​. However, no QTL for whitefly resistance has been previously reported in cassava. High-resolution trait mapping in cassava is a significant and ongoing challenge. This is largely due to cassava's inherent genetic complexity: it is highly heterozygous, predominantly clonally propagated and possesses complex trait inheritance. Nonetheless, metabolomic and transcriptomic studies have suggested potential mechanisms of resistance associated with *A. socialis* ​(Nye et al. 2023; Perez-Fons et al. 2023)​. To pinpoint the underlying genetic loci, genomics studies are crucial, building upon the insights from these omics approaches regarding resistance to *A. socialis*. Identification of loci linked to whitefly resistance would enable the development of molecular markers thereby increasing the efficiency of trait introgression into elite cassava.

The objective of this study was to identify QTL associated with *A. socialis* whitefly resistance in an F_2_ cassava population derived from ECU72, and to develop molecular markers linked to QTL to enable marker-assisted selection for the trait.

## Materials and methods

### Plant material

The AM1588 F_2_ mapping population used in this study was developed by selfing an F_1_ produced from a cross between the whitefly-resistant landrace ECU72 (Bellotti and Arias 2001; Parsa et al. 2015; Atim et al. 2024)​, and the whitefly-susceptible landrace COL2246. ECU72 which is mostly male sterile, is from Ecuador and was used as the female parent, while COL2246 is from Colombia. The crossing blocks were established at the International Center for Tropical Agriculture (CIAT), Palmira, Colombia, where hand pollinations were performed in the field.

**Fig. 1:**
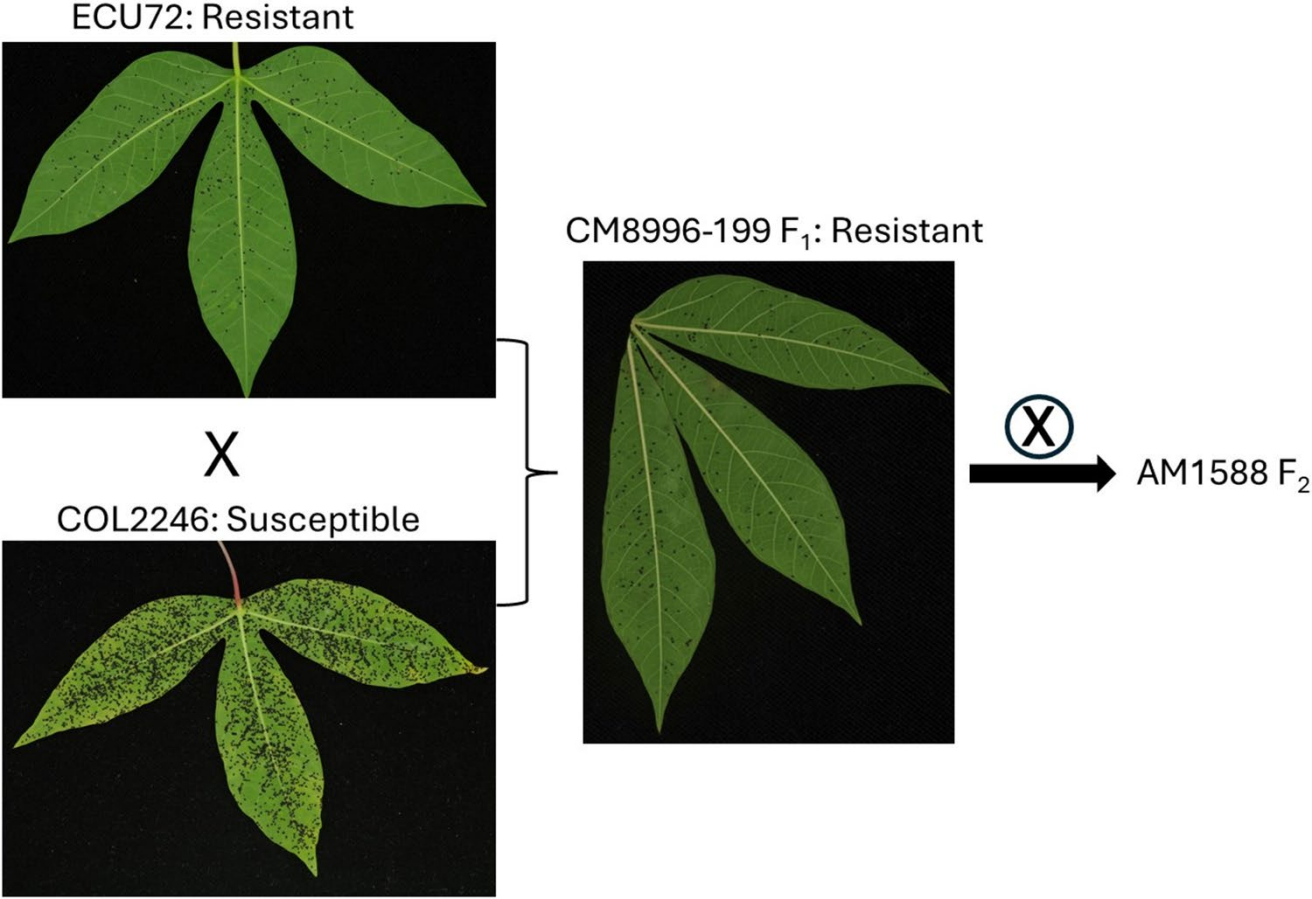
Phenotypes of the two cassava genotypes ECU72 (resistant) and COL2246 (susceptible), and their F_1_ (resistant) 34 days post infestation with *A. socialis*. CM8996-199 was selfed for the AM1588 mapping population development

The F_1_ individual selected for generating the F_2_ mapping population, CM8996-199, exhibited high levels of resistance similar to its resistant parent, ECU72 (Fig. 1) and produced abundant female and male flowers. It was selfed in the 2018–2019 season, resulting in an F_2_ progeny of 183 individuals. These progeny were used for mapping and identification of loci associated with whitefly resistance.

To validate the QTL identified in the AM1588 population, two sets of genetic backgrounds were used. These included other F_2_ populations derived from the ECU72 background, as well as CIAT’s genomic selection training population (GS), consisting of cassava accessions of various pedigrees. Specifically, three F_2_ populations with ECU72 genetic background were used: GM12202 (a pseudo-F_2_ population derived from CM8996-199 × CM8996-758 with 119 progeny) and two smaller F_2_ populations, AM1620 (70 progeny) and AM1621 (24 progeny), both derived from GM8586 F_1_ [ECU72 × TMS60444 (susceptible African line)]. Additionally, 673 accessions from various families of the GS population, cohort 2–2021, were also used for QTL validation (Table S1).

### Mass rearing of *Aleurotrachelus socialis* (bondar) and phenotyping

A methodology for mass rearing *A. socialis*, developed for glasshouse assays and capable of producing about 7600 whitefly adults per plant was used (Bohorquez-Chaux et al. 2023). Phenotypic evaluations were conducted in a glasshouse over three years: 2020–2021 for F_2_ populations, and in 2023 for the GS population using choice experiments. The plants were propagated in one of three methodologies, from in vitro (AM1588), stakes (AM1588 and GS) and micro-stakes (F_2_ and pseudo-F_2_ validation populations),

F_2_ sexual seeds were planted to obtain mother plants. The mother plants from AM1588 were subsequently used to obtain meristems for in vitro propagation and to produce stakes in field conditions. This allowed us to evaluate potential differences in the phenotyping experiments based on the propagation method. Micro-stakes were obtained from mother plants from the other F_2_ for QTL validation. The infestation was made using the methodology described by Bohorquez-Chaux et al. (2023).

An incomplete randomized block design was used separately for each propagation method and population. For the evaluation of AM1588, respective resistant and susceptible checks [ECU72 as resistant, CM8996-199 F_1_ which is the parent of the population, as well as an infestation/susceptible check (COL1468)] were included in each batch of plants. Large white mesh cages (18 m length × 3 m width × 3 m height) located in a glasshouse were used for the evaluation. Each week three batches of 100 plants (total = 300 plants) with at least five completely opened leaves were infested at the same time. Since the number of genotypes to be evaluated exceeded the capacity of each cage, the number of plants per genotype in every experimental batch was unequal. For AM1588, four plants per genotype were evaluated for each propagation method in eighteen and thirteen experimental batches for plants from in vitro and stakes, respectively. For validation, the F_2_ populations (AM1620 F_2_, AM1621 F_2_ and GM12202 pseudo-F_2_) and GS population were evaluated using the same methodology described for AM1588 F_2_. Thirty-four (34) days after infestation, the two most infested leaves were collected (L1 and L2) and photographs taken in a photo box. Analysis for nymph count (NC) and percentage of the leaf occupied by nymphs (%Area) were made using the Nymphstar automated nymph counting method ​(Bohorquez-Chaux et al. 2023). In total, 12 traits were calculated and used for downstream analyses, including NC and %Area for leaf 1, leaf 2 and BLUPs from both leaves, in the in vitro and stake datasets (2 × 3 × 2). These traits are coded as L1_NC, L2_NC, BLUPs_NC and L1_%Area, L2_%Area and BLUPs_%Area in subsequent text.

### Statistical analysis

The statistical analyses were performed in SAS 9.4 software (SAS Institute Inc., Cary, NC) for Linux using the PROC GLIMMIX (generalized linear mixed models—GLMM) procedure. Correlation between the stake and in vitro datasets was assessed using Pearson correlation (*r*) analysis in the R stats package ​(R Core Team 2023)​. Analysis of variance (ANOVA) were performed to measure the effect of genotypes, leaves (L1 and L2), and propagation methods (stake or in vitro) on the NC and %Area. The statistical model applied for each analysis is defined as follows:

$$y = \, {{\varvec{\upmu}}} \, + \, {\mathbf{G}} \, + \, {\mathbf{rep}} \, + \, {\mathbf{L}} \, + \, {{\varvec{\upvarepsilon}}}$$

where*** y*** represents either the NC or the %Area, **µ** denotes the overall mean effect, **G** captures the effect of different cassava genotypes, **rep** accounts for the different experimental batches,** L** represents the effect of leaf position (i.e., L1 (younger leaf) and L2), and **ε** is the residual effect. All effects were treated as random. Best Linear Unbiased Predictions (BLUPs) for each accession were calculated for the different experiments and used for QTL identification. The phenotypic distributions of scores from the NC and %Area, for stakes and in vitro, were tested for deviations from normality with Shapiro–Wilk tests (Shapiro and Wilk 1965).

Broad sense heritability (H^2^) ​(Cullis et al. 2006)​ was calculated using the following equation:

$${\mathbf{H}}^{{\mathbf{2}}} = {\mathbf{1}} - {\varvec{V}}_{{{\varvec{BLUP}}}} /2{\varvec{\sigma}}^{{\mathbf{2}}}_{{\varvec{g}}}$$

where *V*_BLUP_ is the mean–variance difference of two AM1588 F_2_ individuals based on BLUPs and σ^2^_*g*_ is the genetic variance of these genotypes. A linear mixed-effects model was fitted to estimate the variance components using the R package lme4 (function lmer) ​(Bates et al. 2015).

### Genotyping, SNP analysis and linkage map construction

Total genomic DNA was extracted from fresh young leaf tissues harvested from the AM1588 F_2_ family and parents, according to Doyle and Hortorium (1991)​. RAD sequencing ​(Davey and Blaxter 2010)​ was performed on 183 genotypes of the AM1588 F_2_ family at BGI (China). Data were aligned to the *Manihot esculenta* v6.1 genome (https://phytozome-next.jgi.doe.gov/info/Mesculenta_v6_1) using BWA-MEM algorithm ​(Li and Durbin 2010)​. Single nucleotide polymorphisms (SNPs) were called and filtered using GATK ​(Depristo et al. 2011)​, retaining only biallelic SNPs. To ensure high-quality SNPs, additional filters were applied including no more than 10% missingness, ≥ 5% MAF, quality ≥ 30, as well as a depth ≥ 6, using VCFtools ​(Danecek et al. 2011). Finally, a custom Python script was used to remove non-informative SNPs (e.g., identical loci) and those with high segregation distortion (chi-squared test > 5%). The genetic map was constructed using JoinMap v5 (​Van Ooijen J., 2006)​. Since the AM1588 F_2_ family does not come from two fully homozygous diploid parents (ECU72 and COL2246), heterozygous SNPs were encoded as hkxhk in a.loc file to detect the linkage phases. The results of the phases were then extracted from each linkage group and encoded in an ABH format following this guideline: Initial encoding HH, HK, KK; final encoding to loci with phase {00} A, H, B; final encoding to loci with phase {11} B, H, A. Identical loci and individuals were excluded and linkage groups determined with the independence LOD criterion. The F_2_ map was constructed using regression mapping and distance between markers was calculated using the Kosambi mapping function (K​osambi, 1943)​.

### QTL mapping, candidate gene identification and marker development

QTL mapping was performed using datasets from stakes (6 variables) and in vitro (6 variables) of the AM1588 F_2_ population. This was determined for L1_NC, L2_NC, BLUPs_NC, L1_%Area, L2_%Area and BLUPs_%Area (total of 12 datasets/traits). Mapping was performed using composite interval mapping (CIM) ​(Zeng 1994)​ in WinQTLCart 2.5 ​(Wang et al. 2007)​ with threshold values calculated through permutation tests (1,000 permutations, *α* = 0.05) ​(Churchill and Doerge 1994). CIM analysis was performed with a window size of 10 cM using the standard model (Model 6) with a walk speed of 1 cM and 5 marker cofactors determined by forward and backward regression. Candidate genes within the 2-LOD interval of significant QTL were identified using the *Manihot esculenta* V6 genome (https://phytozome-next.jgi.doe.gov/info/Mesculenta_v6_1).

As a complement, to identify the most significant SNPs associated with whitefly resistance and to compare these results with QTL mapping, a genome-wide association analysis (GWAS) was also performed on the F_2_ population using BLUPs for the 12 traits. For the GWAS, a final marker set of 247,117 was used following filtering (missingness ≤ 0.1, maf ≥ 0.05). Four models were used for the GWAS on the GAPIT R package ​(Wang and Zhang 2021)​: the General Linear Model (GLM), Fixed and Random Model Circulating Probability Unification (FarmCPU), Bayesian-Information and Linkage-disequilibrium Iteratively nested Keyway (BLINK) and the Multi‐Locus Mixed Model (MLMM) ​(Price et al. 2006; Segura et al. 2012; Liu et al. 2016; Huang et al. 2019)​. We incorporated three principal components (PCs) derived from the genotype matrix, along with a kinship matrix to ensure model robustness. We applied a Bonferroni threshold using the formula α/n where α is 0.05 and *n* the number of SNPs, and we also applied a false discovery rate (FDR) correction using the Benjamini–Hochberg method to control false positives in our models (Bonferroni 1936; Benjamini and Hochberg 1995).

Kompetitive allele specific PCR (KASP) assays for significant SNPs were developed by Intertek Group plc, Australia. Three markers were selected from the peak region of the consistent QTL identified from linkage mapping, and the other thirty-three SNPs obtained from the GWAS (total of 36 markers) for assay development. The allele frequencies and marker quality of the significant SNPs were calculated using a metric that calculates the false positive rate (FPR) and false negative rate (FNR) ​(Platten et al. 2019; Mbanjo et al. 2024)​.

### Marker validation

Significant markers were validated on the GM12202 pseudo-F_2_, AM1620 F_2_ and AM1621 F_2_ as well as on the GS training populations described. Leaf punches from the samples were sent to Intertek (Australia) for Low-Density SNP Genotyping (LDSG) using thirty-six KASP markers. These samples included 673 samples from different genetic backgrounds of the GS population as well as a total of 365 samples from ECU72 genetic background, developed for whitefly resistance mapping/validation {AM1588 F_2_ (152), AM1620 F_2_ (70), AM1621 F_2_ (24), GM12202 pseudo-F_2_ (119)}. For analysis, ten whitefly-resistant and susceptible checks were also included as haplotype information for these SNPs was available from previous sequencing. These checks included: ECU72, COL2246, TMS60444, CM8996-199, CM8996-758, GM8586-103, GM8586-64, PER368, PER415 and PER608. Resistant (R), intermediate (I) and susceptible (S) categories were determined for the ECU72 genetic background and the genomic selection training population. This was done by performing a K-means analysis based on the BLUPs for NC and %Area using the base R package 'stats' ​(R Core Team 2023)​. For each population, we assessed the performance of the K-means analysis when the number of cluster (k) varied from 2 to 10. A quality measure of clusters was estimated based on the sum of squares in which the between-cluster sum of squares was divided by the total within-cluster sum of squares, multiplied by 100. This score ranges from 0 – 100%, in which a higher value indicates a better capture of the variance of clusters, however, a value close to 100% could indicate an overfitting of clustering. Finally, using an Elbow method, the optimal number of clusters per population was determined.

## Results

### Whitefly resistance in the F_2_ mapping population

**Fig. 2:**
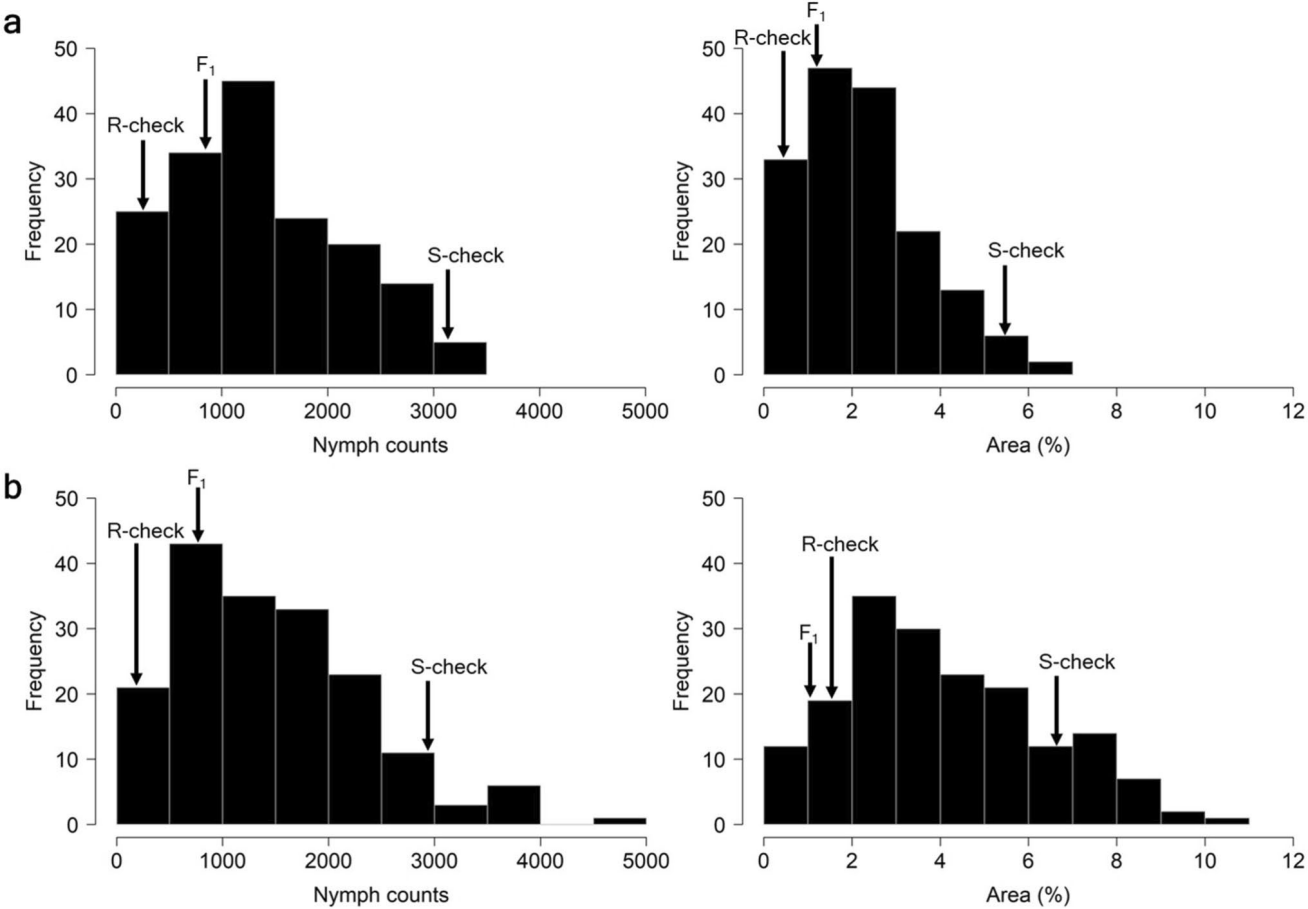
Frequency distribution of AM1588 F_2_ cassava mapping population using **a** stakes and **b** in vitro for nymph counts and percentage of leaf area affected following whitefly infestation (*N* = 183). Arrows indicate the checks (*R* = resistant, *S* = susceptible, *F*_1_ = parent of AM1588 F_2_ mapping population)

The phenotypic distribution for whitefly resistance following infestation confirmed the quantitative nature of the trait. The distributions slightly deviated from a normal distribution according to the Shapiro–Wilk test for normality (*P* = 0.002) (Fig. 2).

The summary statistics for the 12 traits are presented in Table 1. A significant (*P* < 0.0001) positive correlation of *r* = 0.54 and *r* = 0.52 was observed between the datasets from stakes and in vitro, for NC and %Area, respectively (Fig. S1). ANOVA revealed significant differences (*P* < 0.0001) among genotypes, replicates and leaves (Table S2) with a calculated broad-sense heritability (H^2^) of 0.58 and 0.56 for in vitro BLUPs_NC and BLUPs_%Area, and 0.73 and 0.74 for stake BLUPs_NC and BLUPs_%Area, respectively.

**Table 1:** Summary statistics of phenotypic data associated with the 12 traits

Plant material	Trait		Checks		AM1588 F_2_	
		CM8996-199 (F_1_)	ECU72	COL1468	Mean	Range
Stake	BLUPs_%Area	1.30	0.51	5.40	2.29	0.10–6.82
Stake	BLUPs_NC	743.13	402.30	3219.89	1381.50	61.17–3332.63
Stake	L1_%Area	1.22	0.42	6.00	2.41	0.45–6.40
Stake	L1_NC	776.50	209.42	3290.15	1436.45	382.61–3176.30
Stake	L2_%Area	1.28	0.51	4.93	2.15	0.45–5.80
Stake	L2_NC	706.73	301.60	3126.00	1326.31	390.36–3074.73
In vitro	BLUPs_%Area	1.04	1.60	6.10	4.05	0.0422–10.39
In vitro	BLUPs_NC	613.50	605.60	3010.37	1542.00	2.25–4805.33
In vitro	L1_%Area	1.36	1.90	7.17	3.70	0.01–12.2
In vitro	L2_%Area	0.69	1.07	5.00	2.25	0.04–8.97
In vitro	L1_NC	847.50	578.80	3248.07	1840.02	771.97–3425.26
In vitro	L2_NC	378.50	330.85	2510.49	1231.30	469.16–2742.46

L1 = leaf 1, L2 = leaf 2, NC = nymph count

### Genotyping, SNP analysis and map construction

**Fig. 3:**
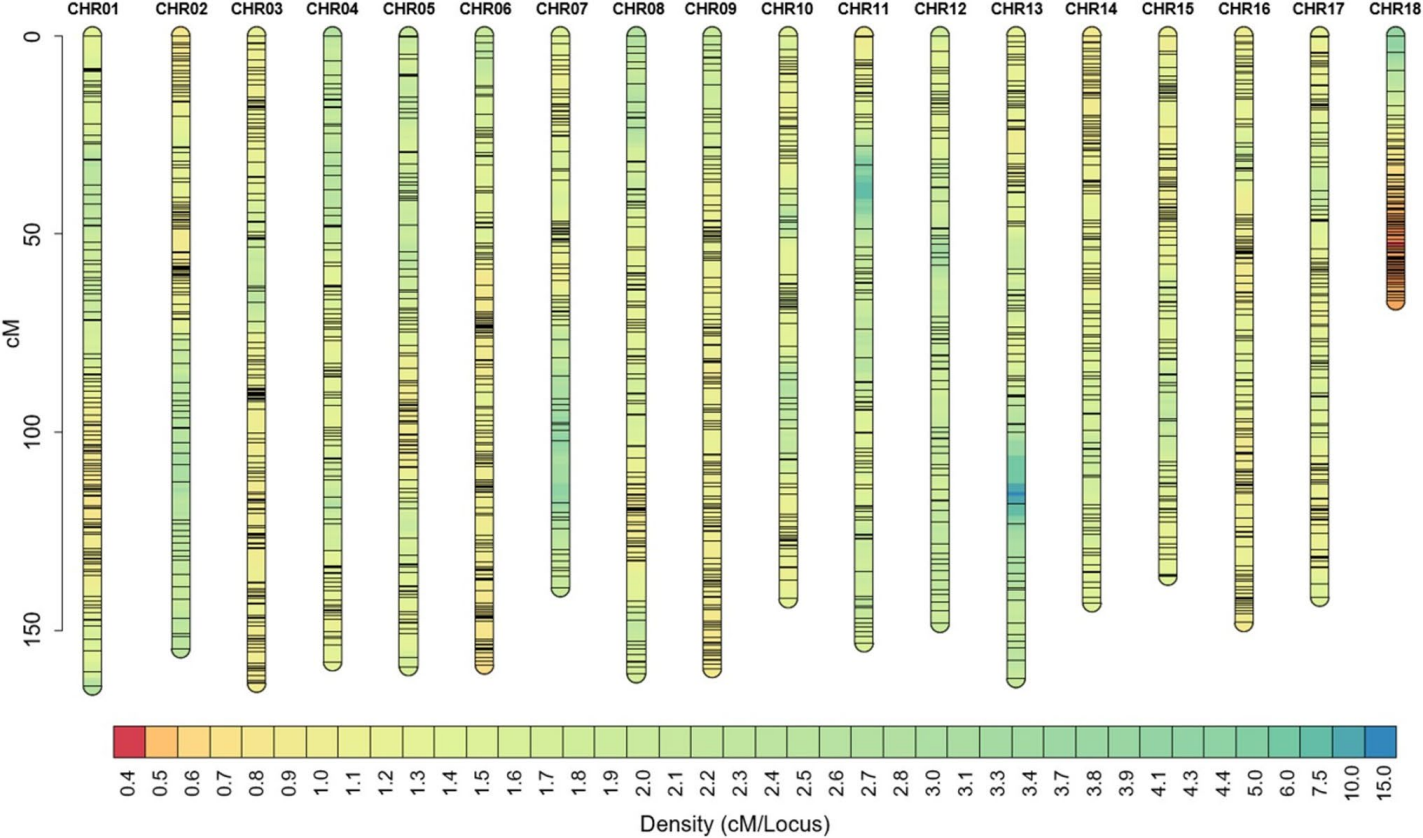
Genetic map of the AM1588 F_2_ population, using 2,017 SNP markers. The horizontal bars on each chromosome represent mapped SNPs, and the scale bar to the left indicates the chromosome length in cM. The bottom bar with the color gradient represents the intra-locus density, denoting distance between markers

A total of 390,234 SNPs were obtained from the RAD sequencing. After filtering, a genetic map was constructed using 2,017 SNPs on 18 chromosomes, with a total length of 2658.2 cM and an average of 112 markers per chromosome. Chromosome 18 was significantly shorter (66 cM) compared to the other chromosomes, despite relatively high density of markers (101) with shorter inter-locus distance, while chromosome 1 was the longest (164.1 cM) (Fig. 3; Table S3).

### QTL identification

**Fig. 4:**
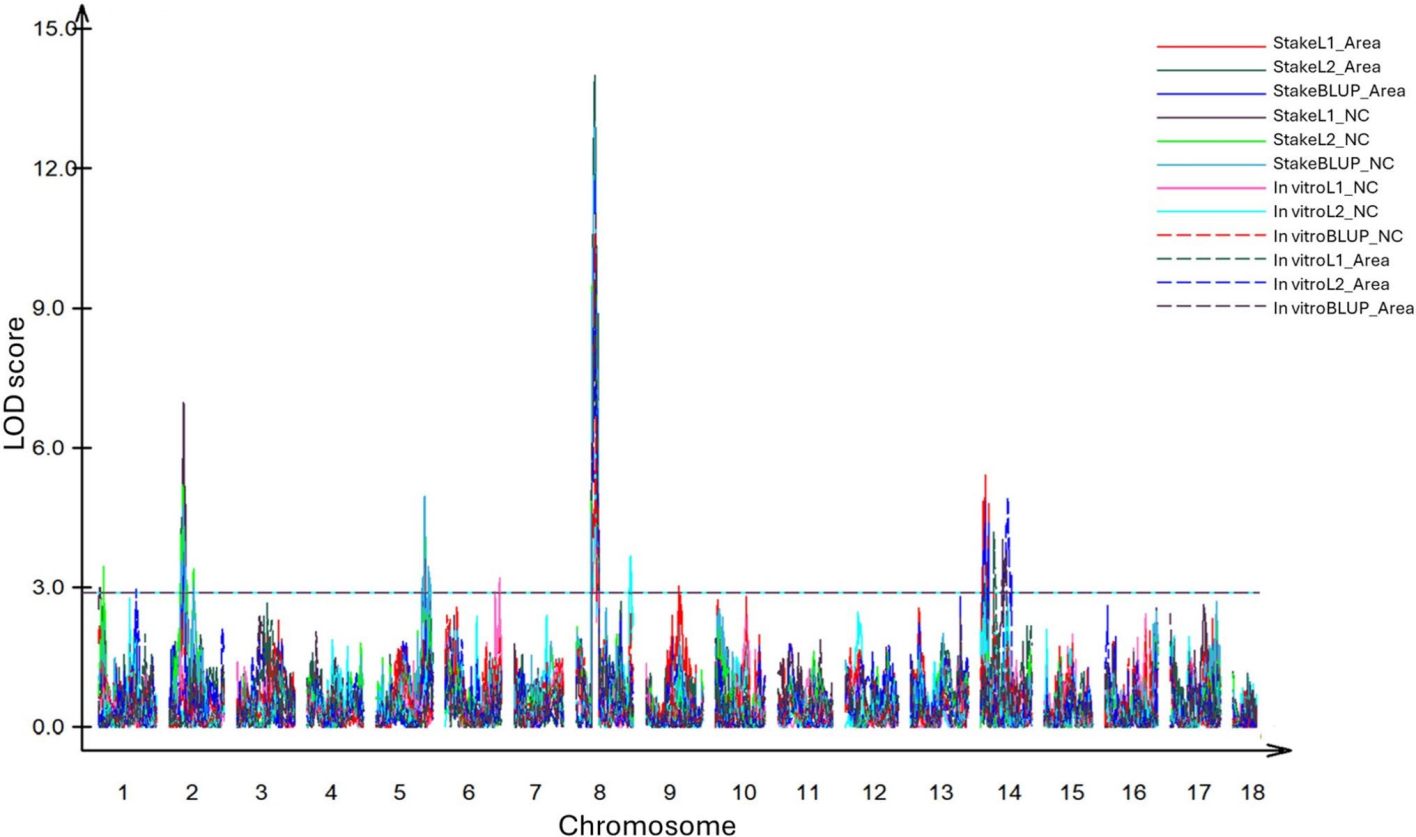
QTL associated with *Aleurotrachelus socialis* whitefly resistance in the AM1588 F_2_ cassava population (*N* = 183)

For the 12 different traits mapped in the AM1588 F_2_ population, QTL were identified in chromosomes 1, 2, 5, 6, 8, 9 and 14 with R^2^ ranging from 0.35%—35.44% (Fig. 4; Table 2). Among these, the QTL on chromosome 8 was the most consistent and with high LOD across all twelve traits (maximum LOD = 14.02), with QTL on chromosomes 2, 5, and 14 also consistent across at least 6 traits. These 4 consistent QTL (2, 5, 8 and 14) also had the highest R^2^ values. Table 1 represents the various QTL for the different traits with their respective LOD scores. Several minor QTL were identified; therefore, the focus was on the consistently overlapping QTL (2, 5, 8 and 14) across traits.

**Table 2:** Quantitative trait loci (QTL) associated with *A. socialis* whitefly in the AM1588 F_2_ cassava population and the corresponding 2-LOD support interval for different traits

Trait	Plant source	*QTL name*	Chr	Peak (cM)	LOD^a^	Additive^b^	Dominant^c^	2-LOD interval (cM)^d^	Right flanking marker (Mb)	Left flanking marker (Mb)	R^2^ (%)^e^
L1_NC	Stake	*MeF2WFly1.1*	1	5.01	3.02	473.00	148.70	0–8.3	C01_4531869	C01_6648455	3.60
L2_NC	Stake	*MeF2WFly1.2*	1	14.41	3.47	344.40	177.28	12.9–15.2	C01_7051287	C01_7477364	2.25
L2_%Area	In vitro	*MeF2WFly1.3*	1	105.31	2.98	− 0.56	− 0.53	104.7–107	C01_26237311	C01_26584494	1.02
L1_%Area	Stake	*MeF2WFly2.1*	2	40.01	2.97	− 0.45	0.40	30–47.8	C02_2390274	C02_5496285	8.49
L2_NC	Stake	*MeF2WFly2.1*	2	38.01	5.24	− 123.92	489.43	36.1–45.5	C02_3110867	C02_4327514	9.22
BLUPs_NC	Stake	*MeF2WFly2.1*	2	39.01	4.88	− 146.97	483.28	36.6–48	C02_3132983	C02_5496285	9.33
BLUPs_%Area	Stake	*MeF2WFly2.1*	2	34.21	3.49	− 0.46	0.33	33.9–36	C02_2709930	C02_3110867	9.75
L2_%Area	Stake	*MeF2WFly2.1*	2	34.21	4.51	− 0.46	0.43	30.9–43.6	C02_2390274	C02_4167810	12.45
L1_NC	Stake	*MeF2WFly2.1*	2	40.01	6.98	− 353.10	487.97	30.2–47.4	C02_2390274	C02_5496285	21.80
BLUPs_NC	Stake	*MeF2WFly2.2*	2	68.61	3.17	− 96.02	397.38	65.6–76.2	C02_9733910	C02_12321797	6.58
L2_NC	Stake	*MeF2WFly2.2*	2	68.61	3.4	− 113.20	397.01	65.5–71.5	C02_9733910	C02_12321797	7.71
BLUPs_%Area	Stake	*MeF2WFly5.1*	5	136.41	2.91	− 0.02	− 0.69	134.5–140.2	C05_25698421	C05_26500914	1.42
L1_%Area	Stake	*MeF2WFly5.1*	5	136.41	3.32	0.01	− 0.85	133.7–140.2	C05_25679518	C05_26500914	1.91
L2_NC	Stake	*MeF2WFly5.1*	5	135.41	4.74	− 34.97	− 449.19	134.5–145.3	C05_25698421	C05_26941212	2.05
L1_NC	Stake	*MeF2WFly5.1*	5	150.81	3.76	122.62	− 335.38	134.1–155.9	C05_25698421	C05_28201769	5.12
L2_%Area	Stake	*MeF2WFly5.1*	5	146.01	2.57	0.38	− 0.48	133.5–148	C05_25679518	C05_27076022	10.54
BLUPs_NC	Stake	*MeF2WFly5.1*	5	147.01	4.98	234.19	− 365.54	135.1–148	C05_25896546	C05_27076022	15.61
L1_NC	In vitro	*MeF2WFly6.1*	6	152.31	3.22	335.78	− 259.01	139.3–154.3	C06_25713854	C06_27159371	10.02
L1_NC	In vitro	*MeF2WFly8.1*	8	54.01	5.64	505.94	181.92	48.4–61.5	C08_5317824	C08_7997296	8.40
L2_NC	In vitro	*MeF2WFly8.1*	8	54.01	6.17	451.50	77.82	42.5–53.9	C08_6640496	C08_8097664	11.75
BLUPs_NC	In vitro	*MeF2WFly8.1*	8	54.01	6.71	475.13	99.46	42.5–61.5	C08_5317824	C08_7997296	12.13
L1_%Area	In vitro	*MeF2WFly8.1*	8	54.01	10.01	1.70	0.31	48.1–61.5	C08_5317824	C08_7997265	18.17
L2_%Area	In vitro	*MeF2WFly8.1*	8	54.01	10.18	1.34	0.14	41.9–63	C08_5317824	C08_7380199	18.43
BLUPs_%Area	In vitro	*MeF2WFly8.1*	8	54.01	11.12	1.48	0.18	53.5–61.5	C08_6640496	C08_7380199	20.18
L1_NC	Stake	*MeF2WFly8.1*	8	52.11	9.62	531.94	− 113.48	43.5–62.6	C08_5725346	C08_7380199	24.13
L1_%Area	Stake	*MeF2WFly8.1*	8	52.11	10.6	1.04	− 0.19	43.7–62	C08_5725346	C08_7380199	27.35
BLUPs_%Area	Stake	*MeF2WFly8.1*	8	52.11	12.16	0.99	− 0.23	42.5–60	C08_5250567	C08_7307520	31.06
BLUPs_NC	Stake	*MeF2WFly8.1*	8	52.11	13.03	588.37	− 68.51	43.5–62.6	C08_5250567	C08_7380199	32.46
L2_NC	Stake	*MeF2WFly8.1*	8	52.11	13.01	570.68	− 77.69	53.3–63.9	C08_5250567	C08_6618913	33.02
L2_%Area	Stake	*MeF2WFly8.1*	8	52.11	14.02	0.99	− 0.22	43.5–61.5	C08_5250567	C08_8097606	35.44
L2_NC	In vitro	*MeF2WFly8.2*	8	152.41	3.7	194.73	− 457.65	147.9–153.7	C08_31471311	C08_32428244	9.87
L1_%Area	Stake	*MeF2WFly9.1*	9	92.91	3.04	-0.49	0.07	87.7–94.5	C09_15940829	C09_18730270	6.22
BLUPs_NC	Stake	*MeF2WFly14.1*	14	13.01	3.28	165.29	− 316.79	12.2–15.7	C14_2692787	C14_4488144	7.98
L1_NC	Stake	*MeF2WFly14.1*	14	13.01	3.55	178.84	− 360.52	12.2–15.7	C14_2692787	C14_4488144	8.33
L2_%Area	Stake	*MeF2WFly14.1*	14	13.01	3.39	0.32	− 0.47	11.5–24.2	C14_2505816	C14_5118096	8.50
BLUPs_%Area	Stake	*MeF2WFly14.1*	14	13.01	4.89	0.42	− 0.59	12.2–14.9	C14_2692787	C14_3068061	12.80
L1_%Area	Stake	*MeF2WFly14.1*	14	13.01	4.81	0.48	− 0.74	1–23	C14_1021098	C14_4844595	14.46
L2_NC	In vitro	*MeF2WFly14.2*	14	75.91	2.79	317.86	− 74.39	74.2–78.7	C14_11842045	C14_9988785	7.35
BLUPs_%Area	In vitro	*MeF2WFly14.2*	14	72.41	4.03	0.87	− 0.20	54.4–72.9	C14_8089554	C14_11400601	9.20
L2_%Area	In vitro	*MeF2WFly14.2*	14	75.91	4.97	0.93	− 0.25	63.9–87.4	C14_8860012	C14_14868789	11.72
L1_%Area	In vitro	*MeF2WFly14.3*	14	37.01	4.21	0.70	1.10	35.1–37.5	C14_6986571	C14_6985846	0.35
BLUPs_NC	In vitro	*MeF2WFly14.3*	14	37.01	2.91	117.12	421.18	36.7–37.5	C14_6986750	C14_6985846	0.40

^a^Logarithm of odds ratios at the position of the peak

^b^Additive effect of QTL

^c^Dominance effect of QTL

^d^The QTL interval on genetic map

^e^Percent of phenotypic variance explained by the QTL

L1 = leaf 1, L2 = leaf 2, NC = nymph count

For chromosome 8 that had overlapping QTL (*MeF2WFly8.1)* across all traits (both from in vitro and stakes), the R^2^ range was 8.4–20.18% for plants from in vitro and 24.13–35.44% for stakes. For chromosome 2, two separate QTL were obtained only from stakes, and none from in vitro data. *MeF2WFly2.1* overlapped for all six traits from stake data (*R*^2^ = 8.49–21.8%), while *MeF2WFly2.2* (*R*^2^ = 6.58–7.71%) was identified in the same position for two traits (leaf 2 and the total BLUPs for nymph count). For chromosome 5 QTL (*MeF2WFly5.1*) which were also only obtained from stake data, the locus for all 6 traits overlapped. The R^2^ range was 1.42–15.61%.

Although the QTL on chromosome 14 had peaks at different positions, there was overlap among traits (both in vitro and stakes) and the R^2^ range was 0.35–11.72% for in vitro and 7.98–14.46% for stakes (Table 2). Overall, the loci from the stake data explained higher phenotypic variation. This is consistent with the broad sense heritability obtained in these experiments, where the 2021 (stakes) assessments had higher H^2^ (0.73 NC; 0.74% Area) compared to the moderate H^2^ in the 2020 (in vitro) assessments (0.58 NC; 0.56%Area).

### GWAS analysis and marker validation

Thirty-three SNPs were identified as significant across the four different models. Only 3 SNPs were consistently significant across all models (Chr08_6483145, Chr08_6512259, and Chr08_6512325), while the rest of the SNPs were identified using only the GLM and/or FarmCPU models (Fig. S2, Tables 3 and S4). The 3 SNPs were within the chromosome 8 peak region from the QTL mapping results. The most significant marker (Chr08_6483145) had an *R*^2^ value of 25% in the mapping population. The 36 markers (33 from GWAS and 3 in the peak region from linkage mapping) converted to KASP assays were tested for applicability in marker-assisted selection, of which 12 did not amplify correctly and therefore were not informative in the analyses. In the ECU72 background (F_2_ mapping and validation populations), markers 1–18 (Table 3) indicated additive segregation patterns, with heterozygotes and genotypes carrying the homozygous unfavorable allele, exhibiting intermediate and susceptible resistance levels to whitefly, respectively. Genotypes with homozygous favorable allele were highly resistant. In contrast, for SNP markers 19–24 (Table 3), there was a dominance segregation pattern with no differences in whitefly resistance levels between genotypes carrying the homozygous favorable allele and heterozygotes, while the genotypes with homozygous unfavorable allele were significantly different, exhibiting susceptibility to whitefly.

**Table 3:** Frequencies of the 24 markers obtained using GWAS and QTL mapping. FPR and FNR are included for each marker, and segregation patterns are shown

No	Marker	Approach	Favorable allele	Unfavorable allele	% Hom favorable allele	% Het	% Hom unfavorable allele	FPR (%)	FNR (%)	Segregation pattern
1	Chr08_6483145 (snpME00572)	GWAS and within *MeF2WFly8.1*	C	G	24.5	45.3	30.1	18	25	Additive
2	Chr08_6512259	GWAS and within *MeF2WFly8.1*	T	C	22.5	31.5	44.2	14.7	55.7	Additive
3	Chr08_6512325 (snpME00575)	GWAS and within *MeF2WFly8.1*	C	T	24.8	27.8	43.1	28	36.5	Additive
4	Chr08_6512307	GWAS and within *MeF2WFly8.1*	G	A	14	22.6	63.8	23.3	38.4	Additive
5	Chr08_6512329	GWAS and within *MeF2WFly8.1*	C	T	16.8	23.6	45	16	40.3	Additive
6	Chr08_6640496 (snpME00577)	GWAS and within *MeF2WFly8.1*	G	A	45.9	38.9	15.3	39	9.6	Additive
7	Chr08_6640586	GWAS and within *MeF2WFly8.1*	T	G	22.2	46.2	31	14.3	42.3	Additive
8	Chr08_6825255	GWAS and within *MeF2WFly8.1*	A	G	8.4	48.9	37.2	3.2	26.9	Additive
9	Chr08_3607807*	Only GWAS	C	A	16.3	42.8	40.8	7	32.7	Additive
10	Chr08_3778097*	Only GWAS	C	T	17	49.5	33.5	17.4	26.9	Additive
11	Chr08_7985587	GWAS and within *MeF2WFly8.1*	G	A	24.4	48.9	25.9	15.9	28.8	Additive
12	Chr08_4043469*	Only GWAS	A	T	28.3	7.7	17.1	58	20.6	Additive
13	Chr08_7617732	GWAS and within *MeF2WFly8.1*	C	T	75.5	17.9	6.5	68.8	2	Additive
14	Chr08_3607718*	Only GWAS	T	C	24.2	47.6	27.5	16.2	23	Additive
15	Chr08_3623304*	Only GWAS	T	C	31.3	24.1	36.9	17.6	17.3	Additive
16	Chr08_5627630	GWAS and within *MeF2WFly8.1*	G	C	71.7	20.9	6.7	86	13.3	Additive
17	Chr08_5411780	GWAS and within *MeF2WFly8.1*	G	A	8.5	30.4	60.4	2.6	67.3	Additive
18	Chr08_5725346**	Within *MeF2WFly8.1*	T	A	38.9	41.6	19.1	43.2	11.5	Additive
19	Chr08_5641063	GWAS and within *MeF2WFly8.1*	T	A	36.2	4.9	54.4	36	25	Dominant
20	Chr08_3623233*	Only GWAS	C	T	42.2	24	32.5	19.8	21.1	Dominant
21	Chr08_3607706*	Only GWAS	G	C	47	24.5	25.6	27	25	Dominant
22	Chr08_4078044*	Only GWAS	G	T	66.2	26.4	6.2	52.3	8	Dominant
23	Chr08_2600888	Only GWAS	A	G	6.7	27.1	63.3	13.3	67.3	Dominant
24	Chr08_6618913**	Within *MeF2WFly8.1*	C	T	19	29.5	45.5	13.7	38	Dominant

^*^ and ** represent markers that were identified using only association or linkage mapping, respectively. All other markers were identified using GWAS and were also within the QTL interval. snpME00572, snpME00575 and snpME00577 are Intertek IDs for the respective markers. Hom = homozygous, Het = heterozygous

### Validation of significant SNPs in the ECU72 genetic background and GS training population

For the ECU72 genetic background populations (*N* = 365; AM1588 F_2_, AM1620 F_2_, AM1621 F_2_, and GM12202 pseudo-F_2_), we observed a clustering quality of 80.58% when k = 3. Therefore, the 365 genotypes were classified in three clusters: 171 as resistant (R), 142 as intermediate (I), and 52 as susceptible (S). Of the 6 SNPs that showed a dominant segregation pattern, 4 (Chr08_5641063, Chr08_3623233, Chr08_3607706, and Chr08_6618913) had acceptable percentages of FPR and FNR, and the other 2 had very high percentages of either FPR or FNR (Table 2). Of the 18 SNPs that showed an additive segregation pattern, 14 (Chr08_6483145, Chr08_6512259, Chr08_6512325, Chr08_6512307, Chr08_6512329, Chr08_6640496, Chr08_6640586, Chr08_6825255, Chr08_3607807, Chr08_3778097, Chr08_7985587, Chr08_3607718, Chr08_3623304, Chr08_5725346) had acceptable percentages of FPR and FNR, and the other 4 had very high percentages of either FPR or FNR (Table 3).

For the GS training population, a 78.80% quality measure was calculated when *k* = 3. The 673 genotypes were therefore classified into three clusters: 228 as resistant (R), 310 as intermediate (I), and 135 as susceptible (S).

**Fig. 5:**
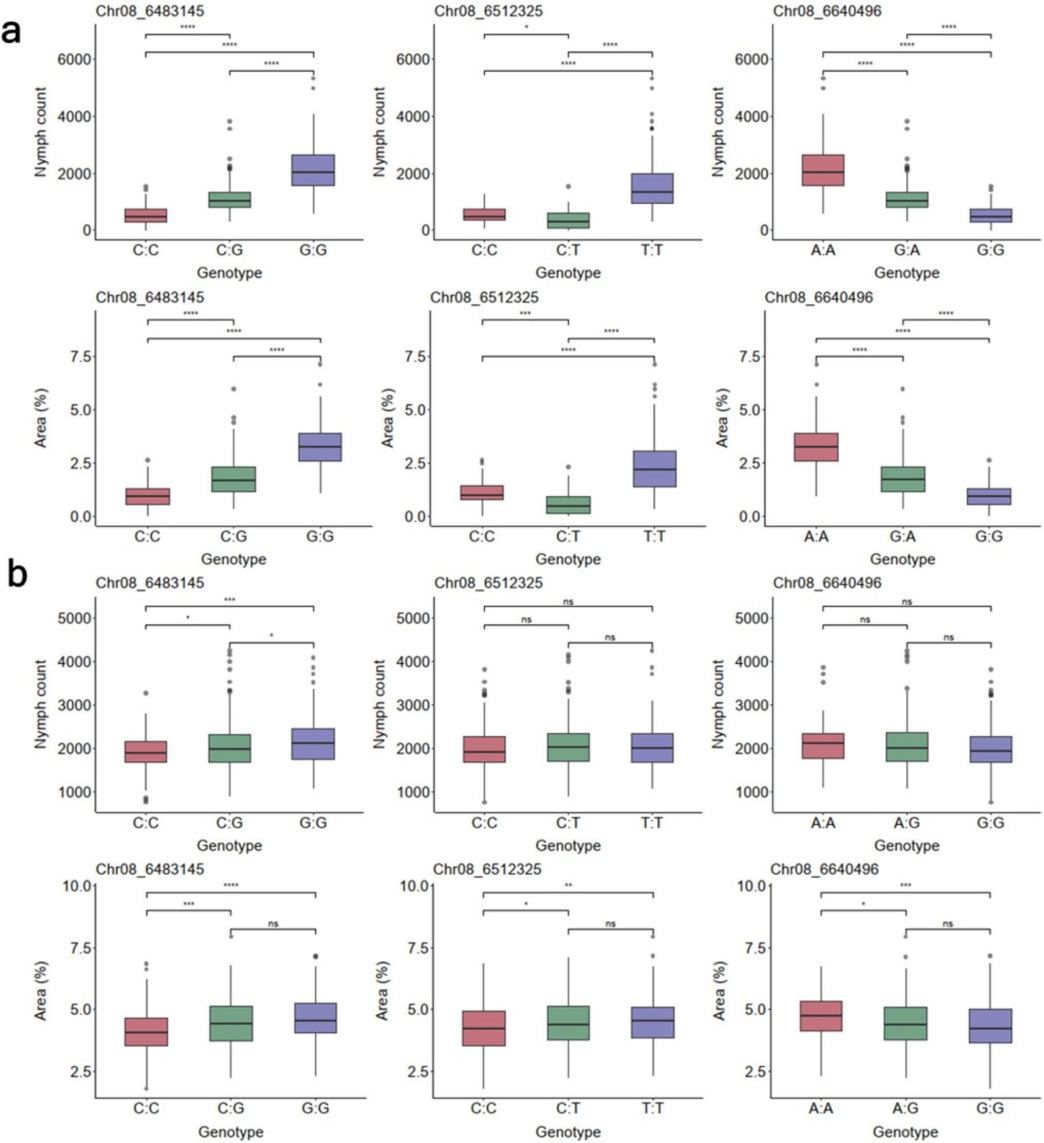
Boxplots displaying the performance of three KASP markers for whitefly resistance in **a**) ECU72 background and **b**) GS training population, for nymph count and percentage leaf area affected. The asterisks indicate levels of statistical significance. *, **, ***, **** significant at *p* ≤ 0.05, *p* ≤ 0.01, *p* ≤ 0.001, *p* ≤ 0.0001, respectively; ns = non-significant (*p* > 0.05)

Based on the validation across the ECU72 background and GS training population, 3 SNPs (Chr08_6483145, Chr08_6512325, and Chr08_6640496), which displayed an additive segregation pattern were the most promising as markers for selection, with Chr08_6483145 best applicable in other genetic backgrounds (Fig. 5). 69.8% of the genotypes evaluated had at least one copy of the favorable allele (C) for the best marker (Chr08_6483145) and were in the R (resistant) or I (intermediate) category. For the other significant markers, Chr08_6512325 (C) and Chr08_6640496 (G), 52.6% and 84.8% of the evaluated genotypes had at least one copy of the favorable allele.

### Candidate gene annotation and functional analysis

Potential candidate genes within the various QTL were identified (Table S5). Table 4 summarizes genes within the loci that have been previously described as involved in host resistance against whiteflies or other insects. *MeF2WFly8.1* harbors 115 annotated genes. Worth highlighting, are genes encoding functions related to resistance against sucking insects, such as those involved in cuticular wax formation, cell wall modification, lignin biosynthesis, antibiosis and association with insect effectors. These genes include MYB106, lectin receptor kinases (LecRLKs), peroxidase 53, strictosidine synthase-like 2 (SSL2), patatin-like phospholipase and trichome birefringence-related gene among others (Table 4). *MeF2WFly14.1* and *MeF2WFly14.2* contain 522 and 400 genes, respectively, with key genes including MYB domain proteins and other genes involved in lignin biosynthesis, trichome development, and immune response against phloem-feeding insects, such as aphids and whiteflies (Table 3). The genes in *MeF2WFly5.1* are 204 and include brassinosteroid insensitive 1 (BRI1), phytocystatin 2 and MYB103 associated with defense mechanisms against insects. The QTL on chromosome 2, *MeF2WFly2.1* and *MeF2WFly2.2* contain 426 and 299 genes, respectively. These genes include MYB domain proteins, ABA-deficient 4, terpenoid synthase and HXXXD-type acyl-transferase family protein, which are involved in cuticular wax biosynthesis, trichome development, response to aphids and whiteflies, and secondary metabolism (Table 4).

**Table 4:** Potential candidate genes associated with whitefly resistance in the identified loci

Plant source	Trait	Approach	SNP	Chr	Gene ID	Gene description	Gene homolog	References	Biological function, crop and insect species
Stake	BLUPs_NC & L1_NC	*MeF2WFly2.1*	Chr02_5122509	2	Manes.02G068600	Abscisic acid (ABA)-deficient 4	ABA4	Guo et al. (2020), Nye et al. (2023)	*Aleurotrachelus socialis, Bemisia tabaci, Acyrthosiphon pisum,* tomato, cassava
Stake	BLUPs_NC & L2_NC	*MeF2WFly2.2*	Chr02_10698625	2	Manes.02G144500	HXXXD-type acyl-transferase family protein	BAHD acyltransferases	Xu et al. (2023), Nye et al. (2023)	Acylation reactions of secondary metabolites, several crops
Stake	BLUPs_NC, L1_%Area, L1_NC & L2_NC	*MeF2WFly2.1*	Chr02_3551813	2	Manes.02G046100	myb domain protein 30	ATMYB30, MYB30	Zhang et al. (2019), Nye et al. (2023)	Cuticular wax biosynthesis*, Malus domestica*
Stake	BLUPs_NC, L1_%Area, L1_NC & L2_NC	*MeF2WFly2.1*	Chr02_3191070	2	Manes.02G041300	myb domain protein 5	ATMYB5, MYB5	Li et al. (2009)	Trichome morphogenesis,* Arabidopsis*
Stake	BLUPs_%Area & L2_%Area	*MeF2WFly2.1*	Chr02_3110867	2	Manes.02G040100	Pentatricopeptide repeat (PPR) superfamily protein		Simon et al. (2020)	*Phyllocolpa sp, Populus trichocarpa*
Stake	BLUPs_NC, L1_%Area, L1_NC & L2_NC	*MeF2WFly2.1*	Chr02_2530211	2	Manes.02G032800	sterol-4alpha-methyl oxidase 1–1	ATSMO1, ATSMO1-1, SMO1-1	Behmer et al. (2013)	*Myzus persicae, Nicotiana tabacum, Phaseolus vulgaris*
Stake	BLUPs_NC, L1_%Area, L1_NC & L2_NC	*MeF2WFly2.1*	Chr02_2718591	2	Manes.02G035200	Terpenoid synthase superfamily protein		Boncan et al. (2020)	Responses to herbivory, several crops
Stake	BLUPs_NC & L1_NC	*MeF2WFly2.1*	Chr02_4402369	2	Manes.02G059400	Thioredoxin superfamily protein		Sytykiewics et al. (2020)	*Rhopalosiphum padi & Metopolophium dirhodum,* maize
Stake	BLUPs_NC, L2_%Area & L1_NC	*MeF2WFly5.1*	Chr05_27223321	5	Manes.05G196700	PHYTOCYSTATIN 2	AtCYS2, CYS2	Martinez et al. (2016)	Hemiptera, Acari, and several crops
Stake	L1_NC	*MeF2WFly5.1*	Chr05_27883494	5	Manes.05G203900	WRKY DNA-binding protein 2	ATWRKY2, WRKY2	Tang et al. (2021)	*Ostrinia furnacalis,* maize
Stake	L1_NC	*MeF2WFly5.1*	Chr05_28074549	5	Manes.05G205700	myb domain protein 103	MYB103, MYB80, MS188	Ohman et al. (2013)	syringyl lignin biosynthesis*, Arabidopsis*
Stake	BLUPs_NC, L2_%Area & L1_NC	*MeF2WFly5.1*	Chr05_26523004	5	Manes.05G192200	Transducin/WD40 repeat-like superfamily protein		Guerriero et al. (2015)	Cell wall biosynthesis*, Arabidopsis*
Stake	L1_NC	*MeF2WFly5.1*	Chr05_27867256	5	Manes.05G203800	BRASSINOSTEROID INSENSITIVE 1 (BRI1)	BRI1	Prince et al. (2014)	*Myzus persicae, Arabidopsis*
Stake & In vitro	All traits	GWAS	Chr08_4043469	8	Manes.08G043700	Peroxidase 53-Related		Singh et al. (2013), Nye et al. (2023)	Elicited by *Apis craccivora & B. tabaci, A. socialis*, Cotton, tomato, cowpea, cassava
Stake & In vitro	All traits	*MeF2WFly8.1*	Chr08_5725346	8	Manes.08G055900	strictosidine synthase-like 2	SSL2	Gu et al. (2023)	Monoterpenes alkaloids synthesis, Maize
Stake & In vitro	All traits	GWAS	Chr08_3778097	8	Manes.08G041400	Dirigent Protein 20-Related	DIR proteins	Pei et al. (2023)	Lignan biosynthesis, *Phryma leptostachya*
Stake & In vitro	All traits	GWAS	Chr08_4078044	8	Manes.08G044000	Protein Trichome Birefringence-related	TBL	Sun et al. (2020)	Modify cell wall properties through O-acetylation,* Arabidopsis*
Stake & In vitro	All traits	GWAS	Chr08_3778097	8	Manes.08G041800	Protein STICHEL-Like 2	STICHEL	Ilgenfritz et al. (2003)	Trichome branch number,* Arabidopsis*
Stake & In vitro	All traits	*MeF2WFly8.1*	Chr08_5363907	8	Manes.08G053200	Plant invertase/pectin methylesterase inhibitor superfamily	PME	Silva-Sanzana et al. (2019)	*Myzus persicae, Arabidopsis*
Stake & In vitro	All traits	GWAS	Chr08_6483145	8	Manes.08G058400	Haloacid dehalogenase-like hydrolase (HAD) superfamily protein		Yang et al. (2012)	Synthesis of cutin & suberin*, Arabidopsis*
Stake & In vitro	All traits	*MeF2WFly8.1*	Chr08_5725346	8	Manes.08G055700	transthyretin-like protein	TTL	Kyoung et al. (2004)	BRI1 substrate*, Arabidopsis*
Stake & In vitro	All traits	GWAS & *MeF2WFly8.1*	Chr08_6640496, 6,640,586	8	Manes.08G059700	basic transcription factor 3	ATBTF3, BTF3	Wang et al. (2014)	Stress response*, Arabidopsis*
Stake & In vitro	All traits	GWAS	Chr08_6483145	8	Manes.08G058500	C2H2-like zinc finger protein	zinc finger protein ZAT3-like	Benyó et al. (2023)	Cell wall biogenesis,* Arabidopsis*
Stake & In vitro	All traits	*MeF2WFly8.1*	Chr08_5725346	8	Manes.08G055800	calmodulin binding		Yadav et al. (2022)	*Spodoptera sp, Glycine max*
Stake & In vitro	All traits	GWAS	Chr08_5411780	8	Manes.08G054200	IQ-domain 12	IQD12	Levy et al. (2005)	*Myzus persicae, Arabidopsis*
Stake & In vitro	All traits	GWAS	Chr08_6512259, 6,512,307, 6,512,325, 6,512,329	8	Manes.08G058900	Membrane trafficking VPS53 family protein	ATVPS53, HIT1, VPS53	Rodriguez et al. (2017)	*Myzus persicae, Arabidopsis, Solanum tuberosum*
Stake & In vitro	All traits	*MeF2WFly8.1*	Chr08_5725346	8	Manes.08G056000	Patatin-like phospholipase family protein	SDP1	Simiyu et al. (2023)	Regulates lignin biosynthesis*, Arabidopsis*
Stake & In vitro	All traits	GWAS	Chr08_6512259, 6,512,307, 6,512,325, 6,512,329	8	Manes.08G058800	Concanavalin A-like lectin protein kinase family protein		Tang et al. (2020); Sauvion et al. (2004)	*Psyllid, Acyrthosiphon pisum,* tomato, pea
Stake & In vitro	All traits	GWAS	Chr08_5627630, 5,641,063	8	Manes.08G055000	dehydration-induced protein (ERD15)	CID1, ERD15, LSR1	Kariola et al. (2006)	*Erwinia carotovora, Arabidopsis*
Stake & In vitro	All traits	GWAS	Chr08_6825255	8	Manes.08G060300	CDPK-related kinase 1	ATCBK3, ATCRK1, CRK1	Hettenhausen et al. (2016); Santamaria et al. (2018)	*Spodoptera exigua, Aphis glycines, Glycine max.*
Stake & In vitro	All traits	GWAS	Chr08_6483145	8	Manes.08G058000	myb domain protein 106	MYB106, MIXTA-like R2R3-MYB family	Wang et al. (2020); Oshima et al. (2013)	Cuticular wax formation,* Arabidopsis, Eustoma grandiflorum*
Stake	L1_%Area	*MeF2WFly14.1*	Chr14_1314046	14	Manes.14G014100	ETHYLENE-INSENSITIVE3-like 3	AtEIL3, ATSLIM, EIL3, SLIM1	He et al. (2017)	Stress response*, Arabidopsis*
Stake	L1_%Area	*MeF2WFly14.1*	Chr14_2200538	14	Manes.14G026300	myb domain protein 19	AtMYB19, MYB19	Wang et al. (2017)	*Macrosiphoniella sanborni, Chrysanthemum morifolium*
Stake	All traits	*MeF2WFly14.1*	Chr14_2863792	14	Manes.14G034400	myb domain protein 42	AtMYB42, MYB42	Zhong et al. (2008); Geng et al. (2020)	Lignin biosynthesis during secondary cell wall formation*, Arabidopsis*
In vitro	BLUPs_%Area & L2_NC	*MeF2WFly14.2*	Chr14_9958663	14	Manes.14G115200	myb domain protein 82	AtMYB82, MYB82	Liang et al. (2014)	Trichome development*, Arabidopsis*
In vitro	BLUPs_%Area & L2_NC	*MeF2WFly14.2*	Chr14_11400601	14	Manes.14G128300	plant natriuretic peptide A	PNP-A	Patané et al. (2022)	Effector of* Bemisia tabaci, Arabidopsis*
Stake	All traits	*MeF2WFly14.1*	Chr14_2863792	14	Manes.14G035800	RPM1 interacting protein 2	RIN2	Lee et al. (2015)	Immune responses*, Arabidopsis*
Stake	All traits	*MeF2WFly14.1*	Chr14_2863792	14	Manes.14G035200	SKU5 similar 2	SKS2	Chen et al. (2023)	Root cell wall formation*. Arabidopsis*
In vitro	L2_NC& L2_%Area	*MeF2WFly14.2*	Chr14_11842022	14	Manes.14G133300	Tetratricopeptide repeat (TPR)-like superfamily protein		Zhou et al. (2021)	Disease resistance, tomato
In vitro	L2_NC& L2_%Area	*MeF2WFly14.2*	Chr14_9821251	14	Manes.14G114400	xyloglucan endotransglycosylase 6		Divol et al. (2007); Nye et al. (2023)	*Aleurotrachelus socialis, Myzus persicae,* Celery*, Arabidopsis,* cassava

L1 = leaf 1, L2 = leaf 2, NC = nymph count

## Discussion

### Phenotyping and genetic control of whitefly resistance

The high-throughput and semi-automated screening method for assessing whitefly resistance responses—Nymphstar ​(Bohorquez-Chaux et al. 2023)​ enabled efficient phenotyping of the populations used in this study. The phenotypic distribution of the evaluated traits were slightly skewed in the AM1588 F_2_ mapping population, with transgressive segregation observed in the direction of resistance. The correlation between the two datasets of plants derived from in vitro (2020) and those from stakes (2021) was *r* = 0.54 for nymph counts, and* r* = 0.52 for the percentage of leaf area occupied by nymphs. Broad sense heritability estimates (0.57–0.75) suggested a significant proportion of whitefly resistance is genetically controlled.

The first linkage map of cassava was published by ​Akano et al., (2002​), using RFLPs and SSRs, followed by SNP-based maps ​(​Masumba et al. 2017; Nzuki et al. 2017; Garcia-Oliveira et al. 2020)​. These maps were generated from bi-parental families with contrasting parents for different traits including CMD, CBSD and cassava green mite (CGM). This study represents the first report of a linkage map from a cassava population generated from selfing (CM8996-199 × CM8996-199 = AM1588 F_2_, 183 individuals) and the first genetic mapping for resistance to *A. socialis* whitefly in cassava. Using high throughput phenotyping and genetic mapping, we identified QTL associated with nymph count and percentage of leaf area occupied by nymphs, on chromosomes 1, 2, 5, 6, 8, 9 and 14, in cassava.

Whitefly resistance in cassava appears to be quantitatively controlled, as evidenced by multiple QTL identified in this population, indicating that the trait is controlled by several genes. The stable QTL on chromosome 8 (*MeF2WFly8.1*), which explained up to 35.44% of the phenotypic variance holds the highest potential for introgression into elite cassava lines due to its consistent effect across traits. Interestingly, *MeF2WFly8.1* co-localizes with the QTL underlying resistance to CGM in genetically diverse cassava panels (Ezenwaka et al. 2018; Rabbi et al. 2022) and may be a good target for multi-pest resistance breeding in cassava. Among the detected loci, we focused our attention on QTL on chromosomes 2, 5, 8 and 14, as they were stable across multiple traits, and explained relatively high proportion of phenotypic variation.

### Cassava defense mechanisms against whiteflies

Whiteflies and other phloem-feeding insects manipulate plant physiology to their advantage, altering host metabolism, reducing photosynthetic efficiency, and inducing structural modifications in plant tissues ​(Thompson and Goggin 2006)​. In response, cassava has evolved multiple resistance mechanisms, broadly classified into antibiosis, antixenosis, and hormonal regulation, each mediated by specific genetic pathways.

### Antibiosis

One of the key mechanisms identified in this study is antibiosis, whereby plants produce secondary metabolites and other compounds that disrupt insect growth and development, and form barriers that defend the plant from subsequent insect attack (Li et al. 2022). Plant lectins, such as ConA-like lectins, were identified within *MeF2WFly8.1* and play an essential role in plant immunity by disrupting the insect digestion and impairing development (Sauvion et al. 2004; Tang et al. 2020). Similarly, peroxidase 53 contributes to whitefly resistance by triggering the reactive oxygen species (ROS)-mediated localized cell death, creating a barrier against further infestation (Singh et al. 2013; Nye et al. 2023). Some enzyme inhibitors including phytostatin 2, block pest digestive proteases, reducing their ability to extract nutrients (Martinez et al. 2016). These findings reinforce the hypothesis that cassava uses protein-based defenses to reduce the survival of whitefly nymphs and limit population expansion.

At the biochemical level, jasmonic acid (JA) signaling pathway enhances plant defense responses. Patatin-like phospholipase proteins identified in *MeF2WFly8.1* have been reported to increase JA accumulation, deterring insect feeding (Canonne et al. 2011; Simiyu et al. 2023). In addition, flavonoids and phenolics regulated by HXXXD-type acyl-transferase proteins (Xu et al. 2023) identified within *MeF2WFly2.2*, also contribute to plant resistance by disrupting insect physiology. Volatile organic compounds (VOCs) synthesized by terpenoid synthases, identified within *MeF2WFly2.1*, may contribute to cassava’s ability to deter whiteflies and in field conditions, attract signal predatory insects (Boncan et al. 2020). Furthermore, the presence of strictosidine synthase-like 2 (SSL2) gene in *MeF2WFly8.1* suggests that cassava’s resistance mechanisms include monoterpene alkaloid biosynthesis, which provide chemical defense against herbivores and pathogens in other plant species like maize (Gu et al. 2023). Together, these metabolic processes highlight cassava’s ability to actively manipulate its chemical environment to resist whitefly infestations.

### Antixenosis

Beyond biochemical defenses involved in antibiosis, cassava also relies on structural barriers (antixenosis), preventing insect feeding and egg-laying (Bellotti and Arias 2001). These barriers include waxes, cell wall modifications, changes in cutin or suberin, and the formation of trichomes. Lignin biosynthesis is a well-documented resistance mechanism in plants against sap-sucking insects. Metabolomics and transcriptomic studies have shown that cell wall reinforcement and changes in lignin content contribute to resistance against pests such as *A. socialis* in cassava (Perez-Fons et al. 2019; Nye et al. 2023). Interestingly Ferguson et al. (2023) found evidence for involvement of the lignin pathway in resistance to CBSD. Notably, key genes responsible for lignin biosynthesis were located within CBSD resistance QTL. This aligns with earlier findings by Amuge et al. (2017), who demonstrated that these lignin biosynthesis genes were upregulated in CBSD-resistant cassava when challenged with the Ugandan cassava brown streak virus. Consequently, genes involved in the lignin pathway hold significant promise as targets for breeding efforts aimed at developing cassava varieties resistant to both CBSD and whiteflies. In this study, MYB domain proteins involved in plant cuticle formation, trichome development and lignin biosynthesis which are crucial for plant defense were also identified in the QTL. Specific examples are MYB106 that regulates cuticular wax formation (Oshima and Mitsuda 2013; Wang et al. 2020), reported in loci associated with CGM severity (Ezenwaka et al. 2018; Rabbi et al. 2022) and MYB103 involved in lignin deposition and secondary cell wall reinforcement (Ohman et al. 2013), which is related to resistance against *A. socialis* whitefly in cassava (Nye et al. 2023). Notably, our lead SNP for whitefly resistance (Chr08_6483145) lies approximately 74 kb downstream of S8_6409580, the top marker for CGM resistance in Rabbi et al. (2022), suggesting that this region may harbor gene(s) conferring broad-spectrum resistance. Other MYB domains (19, 42 and 82) were identified, associated with lignin biosynthesis, secondary cell wall thickening, trichome development and aphid resistance (Zhong et al. 2008; Liang et al. 2014; Wang et al. 2017; Geng et al. 2020). Additional genes associated with plant defense against insects through cell wall structure modifications are described in Table 3 (Ilgenfritz et al. 2003; Divol et al. 2007; Yang et al. 2012; Behmer et al. 2013; Guerriero et al. 2015; Silva-Sanzana et al. 2019; Sun et al. 2020; Benyó et al. 2023; Nye et al. 2023; Pei et al. 2023).

Trichomes play a role in resistance to pests such as whiteflies, though their role varies by plant species. For example, in tomato and Nicotiana, glandular trichomes producing acyl sugars are associated with resistance to the whitefly *Bemisia argentifolii* (Liedl 1995), while cassava studies show no strong correlation between trichome density and whitefly resistance (Parsa et al. 2015; Pastório et al. 2023). Genes involved in trichome formation were identified in this study, however from observation of our populations, we did not note any correlation between genotypes that had visible trichomes, with high levels of whitefly resistance.

### Hormonal signaling

Hormonal regulation is another critical component of whitefly resistance, orchestrating defense responses at a systemic level through abscisic acid (ABA), brassinosteroids (BRs), and ethylene (ET). ABA-deficient mutants lack sufficient callose deposition, making them more susceptible to aphids (*Acyrthosiphon pisum*) and whiteflies (*Bemisia tabaci*) ​(Guo et al. 2020; Nye et al. 2023). Brassinosteroid signaling, mediated by brassinosteroid insensitive 1 (BRI1) and its co-receptor BRI1-associated kinase 1 (BAK1), supports plant growth and pattern-triggered immunity (PTI) restricting herbivore success, as seen in the green peach aphid's (*Myzus persicae*) interaction with Arabidopsis (Prince et al. 2014). Other hormone signaling genes identified include ethylene-insensitive3 (EIN3) transcription factors which enhance defense gene expression against herbivores through ethylene signaling ​(He et al. 2017)​. This pathway interacts with JA signaling, inducing secondary metabolite production and ROS accumulation. The synergistic regulation of ABA, BRs, and ET provides cassava with a multi-layered resistance system, reinforcing physical barriers, metabolic defenses, and signaling cascades that deter whitefly infestation and feeding. Overall, the QTL identified in the study harbor several key genes that potentially contribute to increased levels of resistance against whiteflies in cassava by enhancing various plant defense mechanisms.

### Development and validation of KASP markers for breeding

KASP markers have been developed and implemented for multiple traits in cassava. These include markers for CMD, dry matter content, total carotenoid content, and hydrogen cyanide concentration ​(Esuma et al. 2022; Rabbi et al. 2022; Kanaabi et al. 2024; Mbanjo et al. 2024)​. These markers have proven invaluable for accelerating genetic gains and facilitating precision breeding through marker-assisted selection (MAS). In this study, we identified and validated KASP markers associated with whitefly (*A*. *socialis*) resistance in cassava. The study primarily focused on *A. socialis*, however, the whitefly resistance source (ECU72) demonstrates resistance not only against *A. socialis,* but also against various cryptic species of *Bemisia tabaci* (Omongo et al. 2012; Atim et al. 2024)*,* with likely similar resistance mechanisms. The validation process involved individuals from multiple genetic backgrounds including progeny derived from ECU72 and other genetic backgrounds in the genomic selection training population of the cassava breeding program at CIAT. Three SNPs displayed the highest association with whitefly resistance, with Chr08_6483145 showing the strongest and most consistent association with whitefly resistance, demonstrating high potential for marker-based breeding applications across diverse genetic backgrounds. The utility of these SNPs for marker-assisted selection should be validated in segregating populations from additional genetic backgrounds in other cassava breeding programs, as they show great potential for the genetic enhancement of whitefly resistance in the global cassava community. As more genomic tools and resources are refined for cassava, marker-assisted breeding will significantly improve genetic gains for vital agronomic and quality traits.

## Conclusion

In this study, we employed linkage and association mapping to identify genetic loci conferring whitefly resistance in a cassava F_2_ population. Among the identified QTL, *MeF2WFly8.1* demonstrated stable expression and accounted for up to 35.44% of the observed phenotypic variance. This stability across various genetic backgrounds highlights its significant potential for enhancing whitefly resistance in cassava. Markers applicable in selection were developed and validated, with Chr08_6483145 emerging as the most significant. This marker shows great promise for use in marker-assisted selection (MAS) to improve whitefly resistance in different cassava genetic backgrounds.

## Supplementary Information

Below is the link to the electronic supplementary material.

Supplementary file1 (XLSX 1525 KB)

Supplementary file2 (DOCX 702 KB)

## Data Availability

Data is provided in Electronic Supplementary Material. The leaf images used for phenotyping the mapping population are uploaded on CassavaBase (https://cassavabase.org/).
