# Supplementary material for: Genetic mapping and validation of QTL for whitefly resistance in cassava (Manihot esculenta Crantz)
Source: Theor Appl Genet. 2025 Jun 24;138(7):160. doi: 10.1007/s00122-025-04949-1 (PMC12187898; doi:10.1007/s00122-025-04949-1)
Supplement: Supplementary file 2 — Supplementary file2 (DOCX 702 KB) [file 122_2025_4949_MOESM2_ESM.docx]

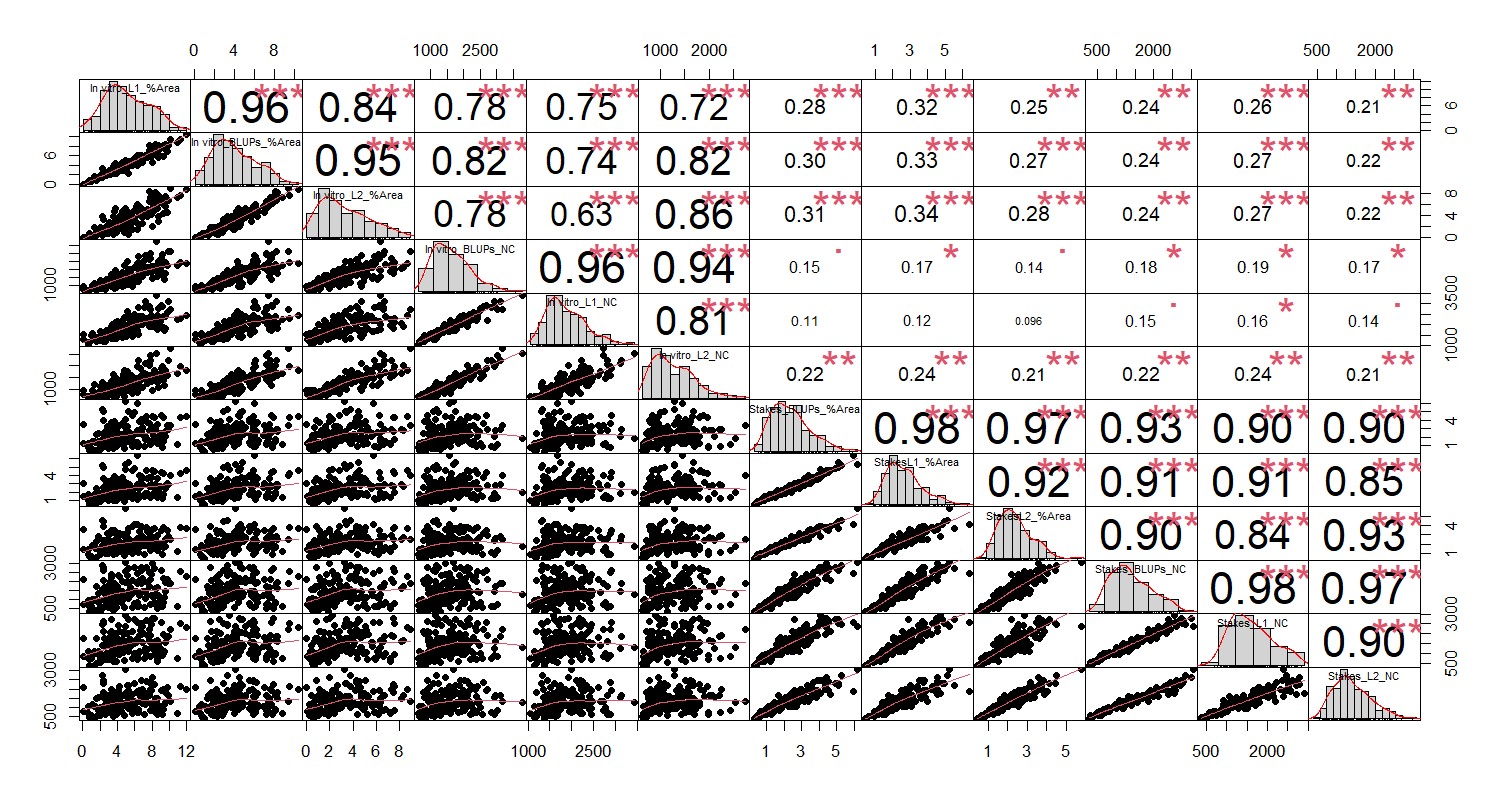


**Fig. S1** Correlation among different traits used in mapping


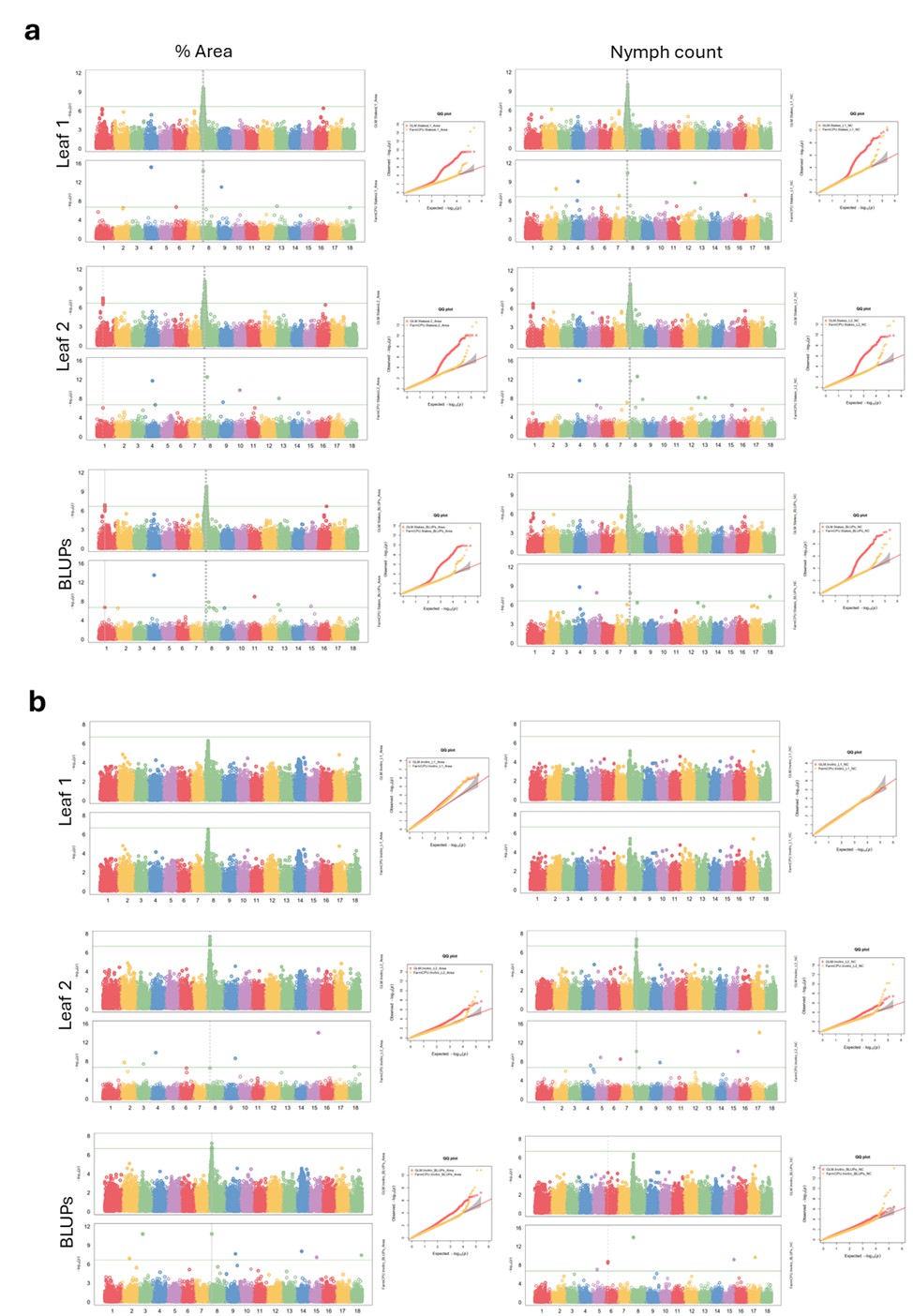


**Fig. S2** Manhattan and respective QQ plots for GWAS of 12 traits using GLM and FarmCPU models in plants propagated from a) stakes and b) in vitro. The horizontal continuous line represents the Bonferroni threshold
